# The importance of chronic conditions for potentially avoidable hospitalizations among non-Hispanic Black and non-Hispanic White older adults in the US: a cross-sectional observational study

**DOI:** 10.1186/s12913-022-07849-y

**Published:** 2022-04-09

**Authors:** Terese Sara Høj Jørgensen, Heather Allore, Miriam R. Elman, Corey Nagel, Ana R. Quiñones

**Affiliations:** 1 Department of Family Medicine, Oregon Health & Science University, Portland, OR USA; 2 Section of Social Medicine, Department of Public Health, University of Copenhagen, Gothersgade 160, 3, 1123 København K, Copenhagen, Denmark; 3 Section of Geriatrics, Department of Internal Medicine, Yale School of Medicine, New Haven, CT USA; 4 Department of Biostatistics, Yale School of Public Health, New Haven, CT USA; 5 School of Public Health, Oregon Health & Science University/Portland State University, Portland, OR USA; 6 College of Nursing, University of Arkansas for Medical Sciences, Little Rock, AR USA; 7 Department of Health Policy and Management, College of Public Health, University of Arkansas for Medical Sciences, Little Rock, AR USA

**Keywords:** Potentially avoidable hospitalizations, Race/ethnicity, Chronic conditions, Machine learning methods

## Abstract

**Background:**

Non-Hispanic (NH) Black older adults experience substantially higher rates of potentially avoidable hospitalization compared to NH White older adults. This study explores the top three chronic conditions preceding hospitalization and potentially avoidable hospitalization among NH White and NH Black Medicare beneficiaries in the United States.

**Methods:**

Data on 4993 individuals (4,420 NH White and 573 NH Black individuals) aged ≥ 65 years from 2014 Medicare claims were linked with sociodemographic data from previous rounds of the Health and Retirement Study. Conditional inference random forests were used to rank the importance of chronic conditions in predicting hospitalization and potentially avoidable hospitalization separately for NH White and NH Black beneficiaries. Multivariable logistic regression with the top three chronic diseases for each outcome adjusted for sociodemographic characteristics were conducted to quantify the associations.

**Results:**

In total, 22.1% of NH White and 24.9% of NH Black beneficiaries had at least one hospitalization during 2014. Among those with hospitalization, 21.3% of NH White and 29.6% of NH Black beneficiaries experienced at least one potentially avoidable hospitalization. For hospitalizations, chronic kidney disease, heart failure, and atrial fibrillation were the top three contributors among NH White beneficiaries and acute myocardial infarction, chronic obstructive pulmonary disease (COPD), and chronic kidney disease were the top three contributors among NH Black beneficiaries. These chronic conditions were associated with increased odds of hospitalization for both groups. For potentially avoidable hospitalizations, asthma, COPD, and heart failure were the top three contributors among NH White beneficiaries and fibromyalgia/chronic pain/fatigue, COPD, and asthma were the top three contributors among NH Black beneficiaries. COPD and heart failure were associated with increased odds of potentially avoidable hospitalization among NH White beneficiaries, whereas only COPD was associated with increased odds of potentially avoidable hospitalizations among NH Black beneficiaries.

**Conclusion:**

Having at least one hospitalization and at least one potentially avoidable hospitalization was more prevalent among NH Black than NH White Medicare beneficiaries. This suggests greater opportunity for increasing prevention efforts among NH Black beneficiaries. The importance of COPD for potentially avoidable hospitalizations further highlights the need to focus on prevention of exacerbations for patients with COPD, possibly through greater access to primary care and continuity of care.

**Supplementary Information:**

The online version contains supplementary material available at 10.1186/s12913-022-07849-y.

## Introduction

The United States is currently experiencing demographic changes that reflect a growing proportion of older adults and greater racial/ethnic diversity in the population [1]. At the same time, the prevalence of older adults living with multiple chronic conditions is increasing [2, 3] for which access to medical care and supportive environments is essential to ensure health and quality of life [4]. This development highlights the importance of identifying and ameliorating potential racial/ethnic disparities in healthcare utilization in the United States.

In 2008, one in ten hospitalizations in the United States were identified as potentially avoidable [5]. Ambulatory care sensitive conditions are a group of conditions—such as asthma, poor glycemic control, or urinary tract infection—for which hospitalization would not have been necessary if timely outpatient treatment had been provided. Hospitalizations with these conditions have been widely used as an indicator of access and quality of primary healthcare in the community [6–8]. A number of previous studies have described markedly greater rates of potentially avoidable hospitalizations among race/ethnic minority groups compared to non-Hispanic (NH) White adults in the US [8–10]. Chronic conditions have furthermore been identified as important risk factors of potentially avoidable hospitalizations and hospitalization costs [11–16]. Yet, whether and how the burden of chronic conditions impact the risk of potentially avoidable hospitalizations across racial groups still needs to be elucidated. To focus prevention and healthcare planning with the current demographic development in the United States, it is important to identify the most important chronic conditions for hospitalizations and potentially avoidable hospitalizations in diverse racial groups. This information can help guide efforts to connect patients to preventive primary care services and whether to focus on specific patient groups to tackle racial disparity in hospitalizations and potentially avoidable hospitalizations.

This study was undertaken to identify chronic conditions preceding hospitalization and potentially avoidable hospitalizations among NH Black and NH White Medicare beneficiaries.

## Methods

### Data source

We used 2014 Centers for Medicare and Medicaid Services (CMS) claims for participants of the Health and Retirement Study (HRS) linked with sociodemographic data from their most recent, preceding HRS interview. In brief, the HRS is a nationally representative survey of individuals aged ≥ 51 years. The survey has been conducted biennially since 1992 with refreshment samples added every 6 years [17]. HRS survey data was linked to Medicare administrative data for age-eligible fee-for-service beneficiaries. The study protocol was approved by Oregon Health and Science University—Research Integrity Office Institutional Review Board (STUDY00017034).

### Study samples

The selection of participants (*N* = 4,993) for the study of hospitalizations was based on enrollment in fee-for-service Medicare Part A and B in 2014, age ≥ 65 years, being NH White or NH Black, and complete information on contributors including the Chronic Condition Data Warehouse (CCW) algorithms. At least three years of enrollment in the Medicare fee-for-service program was required to identify the conditions based on the CCW algorithms [18, 19]. From this sample, a subset (*N* = 1,120) of the study sample with at least one hospitalization was defined for the study of potentially avoidable hospitalizations. The details of the selection of study participants are available in Supplementary Figure S1.

### Variables

Chronic conditions up to the time of HRS interview were the main exposure variables: hypertension, hyperlipidemia, anemia, rheumatoid arthritis/osteoarthritis, ischemic heart disease, heart failure, chronic kidney disease, diabetes, depression, chronic obstructive pulmonary disease (COPD), fibromyalgia/chronic pain/fatigue, atrial fibrillation, acquired hypothyroidism, Alzheimer’s disease and related disorders/senile dementia (ADRD), stroke/transient ischemic attack, anxiety disorders, osteoporosis, cancer, obesity, asthma, pressure and chronic ulcers, acute myocardial infarction, mobility impairments, substance abuse (both drug and alcohol), multiple sclerosis, spinal injury, hip fracture, autism spectrum disorder, bipolar disorder, hepatitis, HIV and schizophrenia. The chronic conditions were identified from linked Medicare beneficiary files and administrative claims using CCW algorithms [18, 19]. A description of the methodology to ascertain each chronic condition can be found at the CCW website [18, 19].

Sociodemographic factors were identified in HRS data and include: sex (male as reference); race categorized as NH White and NH Black; education as a continuous variable of years in school; and wealth as a continuous variable with increments of $10,000. Wealth was truncated at its 95th percentile ($2,044,220) and any value of wealth above $2,044,220 was given that value. Age as a continuous variable was included from the CMS claim for participants’ first event in 2014.

The outcomes were hospitalizations and potentially avoidable hospitalizations occurring in 2014 CMS claims. Hospitalizations were identified as a binary indicator (no/yes) by all inpatient hospital claims in the Medicare claims data. Potentially avoidable hospitalization was constructed as a binary indicator (no/yes) based on the definition by Segal et al. 2014 specifically developed for Medicare-Medicaid Eligible Beneficiaries [20] and widely used in studies of Medicare claims data [21–24]. Our potentially avoidable hospitalization variable used the classification based on both institutional and non-institutional settings that included the following nine inpatient hospital claims with the 9^th^ revision of International Classification of Disease diagnosis categories: COPD, chronic bronchitis and asthma; congestive heart failure; constipation, fecal impaction, and obstipation; dehydration, volume depletion including acute renal failure and hyponatremia; hypertension and hypotension; poor glycemic control; seizures; urinary tract infection; and weight loss (failure to thrive) and nutritional deficiencies. The ICD-9 codes are available in Supplementary Table S1. Conditions for institutional settings only were not included in this study.

### Statistical models

Descriptive statistics were conducted using means with standard deviations or medians with interquartile ranges (IQR) for continuous variables and frequencies with percentages for categorical variables for each of the outcomes.

Statistical models were constructed in two steps. First, to identify chronic conditions with the highest variable importance in predicting the outcomes, conditional inference random forests were implemented using the R package ‘party’ [25]. This non-parametric, machine learning method uses bootstrap aggregation to create multiple decision trees, each using a random sample of variables as split candidates, and collects their results. Recursive binary partitioning is conducted by the decision trees to explore the relationship between multiple explanatory variables and one outcome. In this process, a decision tree is constructed by testing the null hypothesis of independence between each variable and the outcome. If the hypothesis cannot be rejected, the algorithm is stopped. The variable with the greatest reduction of heterogeneity in the outcome is selected and a binary split of the variable is performed. Each forest was created using 1,500 trees and we repeated the analyses three times with different random seeds to confirm the robustness of results. The number of potential variables to try at each potential split were set to the default (square root of the number of predictors in the model). From these analyses, we obtained a ranking of variable importance in predicting the outcome. The conditional inference random forest differs from the random forest implemented in the R package ‘randomForest’ by 1) being unbiased when predictor variables are of different types and 2) include a conditional permutation importance measure that helps evaluate the importance of correlated predictor variables [26]. Second, the top three chronic conditions identified in the ranking were included in multivariable logistic regression analyses adjusted for sociodemographic factors to identify risk estimates (adjusted odds ratios [aORs]) and 95% confidence intervals (CIs) and quantify the association for each of the two outcomes.

## Results

### Descriptive results

Baseline characteristics for study participants by race and hospitalizations are presented in Table 1. In total, 22.1% of NH White and 24.8% of NH Black beneficiaries had at least one hospitalization. For both racial groups, those with at least one hospitalization were older, more often female, and had a lower median wealth. All chronic conditions were more frequent in individuals of both races with hospitalizations compared to those without.

**Table 1:** Characteristics of respondents by race and hospitalization status

	Non-Hispanic White (*n* = 4,420)				Non-Hispanic Black (*n* = 573)			
Characteristics	Hospitalization (*n* = 978)		No Hospitalization (*n* = 3,442)		Hospitalization (*n* = 142)		No Hospitalization (*n* = 431)	
Age (years), mean (SD)	80.0	(7.5)	78.1	(7.2)	78.5	(7.5)	76.6	(6.7)
Female, n (%)	544	(55.6)	2083	(60.5)	83	(58.5)	286	(66.4)
Wealth (USD^a^), median (IQR)	22.4	(6.9, 61.0)	31.9	(11.4, 78.1)	3.9	(0.1, 13.2)	6.5	(0.6, 18.9)
Education (years), median (IQR)	12.0	(12.0, 15.0)	12.0	(12.0, 16.0)	12.0	(9.0, 12.0)	12.0	(10.0, 14.0)
Chronic Conditions, n (%)^b^								
Hypertension	935	(95.6)	2848	(82.7)	141	(99.3)	393	(91.2)
Hyperlipidemia	887	(90.7)	2820	(81.9)	128	(90.1)	342	(79.4)
Anemia	816	(83.4)	1895	(55.1)	130	(91.5)	312	(72.4)
Rheumatoid arthritis/osteoarthritis	796	(81.4)	2154	(62.6)	105	(73.9)	276	(64.0)
Ischemic heart disease	756	(77.3)	1763	(51.2)	119	(83.8)	239	(55.5)
Heart failure	557	(57.0)	854	(24.8)	98	(69.0)	151	(35.0)
Chronic kidney disease	521	(53.3)	835	(24.3)	94	(66.2)	163	(37.8)
Diabetes	468	(47.9)	1164	(33.8)	98	(69.0)	230	(53.4)
Depression	503	(51.4)	1018	(29.6)	58	(40.8)	121	(28.1)
COPD	458	(46.8)	857	(24.9)	70	(49.3)	87	(20.2)
Fibromyalgia/Chronic Pain/Fatigue	394	(40.3)	838	(24.3)	46	(32.4)	91	(21.1)
Atrial fibrillation	384	(39.3)	573	(16.6)	33	(23.2)	41	(9.5)
Acquired hypothyroidism	359	(36.7)	1057	(30.7)	44	(31.0)	76	(17.6)
Alzheimer’s disease/senile dementia	325	(33.2)	514	(14.9)	64	(45.1)	77	(17.9)
Stroke/transient ischemic attack	323	(33.0)	548	(15.9)	56	(39.4)	80	(18.6)
Anxiety disorders	333	(34.0)	656	(19.1)	30	(21.1)	63	(14.6)
Osteoporosis	318	(32.5)	885	(25.7)	38	(26.8)	77	(17.9)
Cancer	303	(31.0)	656	(19.1)	39	(27.5)	94	(21.8)
Obesity	287	(29.3)	521	(15.1)	51	(35.9)	89	(20.6)
Asthma	226	(23.1)	402	(11.7)	36	(25.4)	69	(16.0)
Pressure and chronic ulcers	219	(22.4)	247	(7.2)	30	(21.1)	49	(11.4)
Acute myocardial infarction	156	(16.0)	167	(4.9)	23	(16.2)	14	(3.2)
Mobility impairments	105	(10.7)	111	(3.2)	23	(16.2)	32	(7.4)

^a^Per 10,000 USD; ^b^Conditions shown where frequency for all cells for individual conditions is ≥ 11 (Not shown: drug, alcohol and substance abuse, multiple sclerosis, spinal injury, hip fracture, autism spectrum disorder, bipolar disorder, hepatitis, HIV and schizophrenia)

*Abbreviations*: *SD* Standard Deviation, *USD* United States Dollars, *IQR* Interquartile range, *COPD* Chronic Obstructive Pulmonary Disease

For the subset of study participants with hospitalizations, baseline characteristics by race and potentially avoidable hospitalizations are presented in Table 2. In total, 21.3% of NH White and 29.6% of NH Black beneficiaries had at least one potentially avoidable hospitalization. Compared to NH White and NH Black respondents without an potentially avoidable hospitalization, those with at least one potentially avoidable hospitalization were slightly older, more often female, and had a lower median wealth. All chronic conditions were more frequent in individuals of both races with potentially avoidable hospitalizations compared to those without, with the exception of rheumatoid arthritis/osteoarthritis in NH Black respondents.

**Table 2:** Characteristics of respondents by race/ethnicity and potentially avoidable hospitalization status

	Non-Hispanic White (*n* = 978)					Non-Hispanic Black (*n* = 142)			
Characteristics	Potentially avoidable Hospitalization (*n* = 208)		No Potentially avoidable Hospitalization (*n* = 770)			Potentially avoidable Hospitalization (*n* = 42)		No Potentially avoidable Hospitalization (*n* = 100)	
Age (years), mean (SD)	81.1	(8.00)	79.7	(7.32)		78.9	(7.62)	78.3	(7.5)
Female, n (%)	123	(59.1)	421	(54.7)		30	(71.4)	53	(53.0)
Wealth (USD^a^), median (IQR)	18.5	(5.6, 43.5)	24.2	(7.0, 63.0)		0.8	(0.0, 9.1)	4.8	(0.2, 14.3)
Education (years), median (IQR)	12.0	(12.0, 14.0)	12.0	(12.0, 15.0)		12.0	(8.2, 12.0)	12.0	(10.0, 13.0)
Chronic Conditions, n (%)^b^									
Hypertension	202	(97.1)	733	(95.2)		42	(100.0)	99	(99.0)
Hyperlipidemia	190	(91.3)	697	(90.5)		40	(95.2)	88	(88.0)
Anemia	185	(88.9)	631	(81.9)		42	(100.0)	88	(88.0)
Ischemic heart disease	167	(80.3)	589	(76.5)		37	(88.1)	82	(82.0)
Rheumatoid arthritis/osteoarthritis	173	(83.2)	623	(80.9)		30	(71.4)	75	(75.0)
Heart failure	157	(75.5)	400	(51.9)		36	(85.7)	62	(62.0)
Chronic kidney disease	141	(67.8)	380	(49.4)		33	(78.6)	61	(61.0)
COPD	141	(67.8)	317	(41.2)		28	(66.7)	42	(42.0)
Diabetes	111	(53.4)	357	(46.4)		33	(78.6)	65	(65.0)
Depression	115	(55.3)	388	(50.4)		20	(47.6)	38	(38.0)
Atrial fibrillation	96	(46.2)	288	(37.4)		14	(33.3)	19	(19.0)
Alzheimer’s disease/senile dementia	83	(39.9)	242	(31.4)		24	(57.1)	40	(40.0)
Acquired hypothyroidism	87	(41.8)	272	(35.3)		18	(42.9)	26	(26.0)
Fibromyalgia/Chronic Pain/Fatigue	76	(36.5)	318	(41.3)		20	(47.6)	26	(26.0)
Stroke/transient ischemic attack	70	(33.7)	253	(32.9)		20	(47.6)	36	(36.0)
Obesity	67	(32.2)	220	(28.6)		16	(38.1)	35	(35.0)
Asthma	67	(32.2)	159	(20.6)		14	(33.3)	22	(22.0)

^a^Per 10,000 USD; ^b^Conditions shown where frequency for all cells for individual conditions is ≥ 11. (Not shown: drug, alcohol and substance abuse, multiple sclerosis, spinal injury, hip fracture, autism spectrum disorder, bipolar disorder, hepatitis, HIV and schizophrenia)

*Abbreviations*: *SD* Standard Deviation, *USD* United States Dollars, *IQR* Interquartile range, *COPD* Chronic Obstructive Pulmonary Disease

### Hospitalizations

**Fig. 1:**
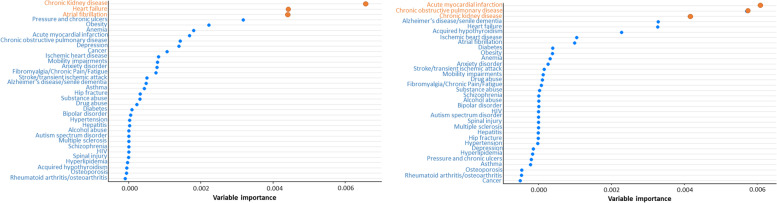
Variable importance rankings of chronic conditions from conditional inference random forests for hospitalizations among 4,420 non-Hispanic White (left) and 573 non-Hispanic Black (right) Medicare beneficiaries

Graphs with variable importance rankings of chronic conditions from conditional inference random forests for hospitalizations are shown in Fig. 1 for NH White (left) and NH Black (right) older adults. The results show that chronic kidney disease, heart failure, and atrial fibrillation were the top three contributors of hospitalizations among NH White and acute myocardial infarction, COPD, and chronic kidney disease were the top 3 contributors of hospitalizations among NH Black beneficiaries. For NH White respondents, these results were identified in all three conditional inference random forests, whereas for NH Black respondents they were identified in two out of three conditional inference random forests. In the last condition inference random forest for NH Black respondents, the top contributors were acute myocardial infarction, COPD, and Alzheimer’s disease and related disorders/senile dementia (results not show).

Table 3 shows results from multivariable logistic regression analyses that included the top three chronic conditions predicting hospitalization in the conditional inference random forest for NH White and NH Black beneficiaries. Chronic kidney disease (aOR: 2.41; 95% CI: 2.06, 2.84), heart failure (aOR: 2.39; 95% CI: 2.02, 2.84), and atrial fibrillation (aOR: 1.99; 95% CI: 1.67, 2.38) were associated with significantly increased odds of hospitalizations among NH White respondents after adjusting for sociodemographic factors. Acute myocardial infarction (aOR: 3.39; 95% CI: 1.60, 7.39), COPD (aOR: 2.79; 95% CI: 1.81, 4.30), and chronic kidney disease (aOR: 2.34; 95% CI: 1.53, 3.61) were associated with significantly increased odds of hospitalizations among NH Black respondents when the model was adjusted for sociodemographic factors.

**Table 3:** Results for hospitalizations by race for the top three chronic conditions from race-specific random forests

	Non-Hispanic White (*N* = 4,420)		Non-Hispanic Black (*N* = 573)	
Chronic conditions	aOR^*^	(95% CI)	aOR^*^	(95% CI)
Chronic kidney disease	2.41	(2.06, 2.84)	2.34	(1.53, 3.61)
Heart failure	2.39	(2.02, 2.84)	-	
Atrial fibrillation	1.99	(1.67, 2.38)	-	
Acute MI	-		3.39	(1.60, 7.39)
COPD	-		2.79	(1.81, 4.30)

^*^All models adjusted for age, sex, net worth, net worth squared, and education

*Abbreviations*: *OR*  Odds Ratio, *CI* Confidence Interval, *MI*  Myocardial Infarction, *COPD*  Chronic Obstructive Pulmonary Disease

### Potentially avoidable hospitalizations

**Fig. 2:**
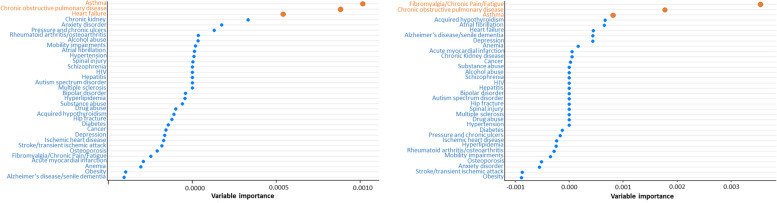
Variable importance rankings from Conditional Inference Random Forests for potentially avoidable hospitalizations among 978 non-Hispanic White (left) and 142 non-Hispanic Black (right) Medicare beneficiaries

The conditional inference random forest of potentially avoidable hospitalizations is shown in Fig. 2 for NH White (left) and NH Black (right) older adults. The results show that asthma, COPD, and heart failure were the top three contributors of potentially avoidable hospitalizations among NH White and fibromyalgia/chronic pain/fatigue, COPD, and asthma were the top three contributors of potentially avoidable hospitalizations among NH Black beneficiaries. These results were identified in all three conditional inference random forest for both races (results not shown).

Table 4 shows the results from multivariable logistic regression analyses that included the top three chronic conditions predicting potentially avoidable hospitalization in the conditional inference random forest for NH White and NH Black older adults. COPD (aOR: 2.35; 95% CI: 1.64, 3.39) and heart failure (aOR: 2.10; 95% CI: 1.46, 3.06) were associated with significantly increased odds of potentially avoidable hospitalizations among NH White when the model was adjusted for sociodemographic factors. COPD (aOR: 2.84, 95% CI: 1.24, 6.77) was the only chronic condition of the top three contributors associated with significantly increased odds of potentially avoidable hospitalizations among NH Black after adjusting for sociodemographic factors.

**Table 4:** Results for potentially avoidable hospitalizations by race for the top three chronic conditions from race-specific random forests

	Non-Hispanic White (*N* = 978)		Non-Hispanic Black (*N* = 142)	
Chronic conditions	aOR^*^	(95% CI)	aOR^*^	(95% CI)
Asthma	1.16	(0.79, 1.69)	1.08	(0.42, 2.72)
COPD	2.35	(1.64, 3.39)	2.84	(1.24, 6.77)
Heart failure	2.10	(1.46, 3.06)	-	
Fibromyalgia/chronic pain/fatigue	-		2.18	(0.98, 4.90)

^*^All models adjusted for age, sex, net worth, net worth squared, and education

Abbreviations: *OR* Odds Ratio, *CI* Confidence Interval, *COPD* Chronic Obstructive Pulmonary Disease

## Discussion

This study showed differences in the importance of chronic disease preceding hospitalization and potentially avoidable hospitalization between NH White and NH Black Medicare beneficiaries in the United States. We suggest that both outcomes permit indirectly assessing the importance of chronic diseases to two different but related healthcare utilization measures. These outcomes reflect access to care for emergent problems in the case of hospitalization and access to continuity of care in the case of potentially avoidable hospitalization. Similar to previous studies, we identified greater rates of having at least one potentially avoidable hospitalization among NH Black older adults compared to NH White older adults [8–10]. Minority groups may experience greater rates of potentially avoidable hospitalizations because they are subject to greater morbidity and mortality from various chronic conditions while at the same time receive lower quality of care and have lower usage of preventive health care services compared to non-minorities [27, 29, 29–33].

To the best of our knowledge, this study is the first to identify whether different chronic conditions are associated with hospitalization and potentially avoidable hospitalizations among NH White and NH Black Medicare beneficiaries. Potentially avoidable hospitalization have been widely used as an indicator of access and quality of primary healthcare in the community, thus, individuals with the chronic conditions found to be associated with avoidable hospitalizations may not have received optimal treatment for a variety of reasons. Asthma and COPD were identified among the top 3 contributors of potentially avoidable hospitalizations for both NH White and NH Black beneficiaries in addition to heart failure in NH White and fibromyalgia /chronic pain/fatigue in NH Black beneficiaries. Yet, asthma was not statistically significantly associated with potentially avoidable hospitalization in either of the groups when adjusted for potential sociodemographic factors. This finding illustrates that, at least to a certain extent, the same chronic conditions are important for predicting potentially avoidable hospitalizations among NH White and NH Black Medicare beneficiaries. Further, our findings that COPD in NH Black and COPD and heart failure in NH White beneficiaries are risk factors of potentially avoidable hospitalizations are in line with previous findings. A study by Dantas et al. 2016 showed that especially chronic conditions of the circulatory and respiratory systems were risk factors for potentially avoidable hospitalizations in Canada. They furthermore found that the number of chronic conditions and the number of influenced body systems were important risk factors for potentially avoidable hospitalizations [13]. An older study by Culler et al. 1998 of a representative sample of Medicare beneficiaries showed that fair/poor health, coronary heart disease, myocardial infarction, and diabetes were associated with increased odds of having an potentially avoidable hospitalization whereas hypertension, stroke, and cancer were not [16]. Our findings of the importance of fibromyalgia /chronic pain/fatigue for potentially avoidable hospitalizations among NH Black beneficiaries may be explained by poor pain treatment among NH Black adults in the United States. A previous review documented extensive racial disparities in pain treatment where minority patients are less likely to have their pain assessed and treated [34]. The results from our study and the previous review, thus, suggest a need for better addressing chronic pain in ambulatory and outpatient care settings for NH Black older adults.

On a final note, our findings highlight a need of increased focus on improving access to and coordinating care for especially NH Black older adults in primary care settings to prevent potentially avoidable hospitalizations. They further highlight that special focus should be placed on patients with COPD among NH Black and patients with COPD and heart failure patients among NH White to potentially prevent potentially avoidable hospitalizations in old age.

This study has several strengths. Through linkage between HRS and CMS, we were able to identify a comprehensive number of chronic conditions to be investigated as potential top contributors of hospitalization and potentially avoidable hospitalizations. We included information on chronic conditions and hospitalizations from CMS, which are not subject to recall bias. We were able to explore our research questions and the impact of these multiple chronic conditions in machine learning models instead of restricting our hypothesis to a priori knowledge. Most clinical risk prediction models are based on regression models, which are limited by only being able to handle a limited number of potential exposure variables in the same model [35]. On the other side, conditional inference random forest is limited by not providing estimates of the relationship between an exposure and outcome variable. A strength of this study is sequencing conditional inference random forest to select the top 3 important chronic conditions then applying them in the logistic regression to estimate adjusted ORs of the associations. Finally, potentially avoidable hospitalizations were measured by a definition developed and widely used for Medicare-Medicaid Eligible Beneficiaries [20–24]. The limitations of the study should also be mentioned. First, the observational nature of the study limits the possibility to draw causal conclusions and that unmeasured confounders may exist. Generalizability of the results to all older Americans may be hampered by restricting the study sample to Medicare beneficiaries with ≥ 3 years of enrollment in the Medicare fee-for-service program at baseline who have participated in the HRS. In this regard, we would like to highlight that individuals with Medicare Advantage are not included, which is critical given the proportion of race/ethnic minorities and other healthcare vulnerable groups who elect managed care over fee-for-service. Furthermore, Table S2 shows sociodemographic characteristics of excluded respondents without 2014 CMS data. Excluded NH white respondents had age and educational level similar to the study sample, whereas they were less often female and had lower wealth. Excluded NH black respondents had similar age to the study sample, whereas they were less often female and had lower wealth and educational level. The selection of the study sample was necessary to identify chronic conditions by the CCW algorithms [18] and sociodemographic factors available in the HRS (Figure S1). The conditional random forest package does not yet allow for survey weights and analysis of a subset meeting inclusion criteria. Furtermore, due to small sample sizes, we were unable to analyze Hispanic participants. We were unable to assess other racial categories—such as Asian Americans—due to the inability to identify additional racial groups in the HRS data. The small number of 142 NH Black older adults with at least one hospitalization, of which 42 have a potentiallty avoidable hospitalization, has low power for estimating the associations. Thus, the findings are exploratory and should be confirmed in futute studies with larger sample before conclusions are drawn. We modeled all cause hospitalization and not causes as a direct results of pre-existing chronic conditions. A final concern is that the findings from the conditional inference random forest of hospitalization were not stable as acute myocardial infarction, COPD, and chronic kidney disease were identified as the top 3 contributors of hospitalizations among NH Black in the first two analyses with different seeds, whereas Alzheimer’s disease and related disorders/senile dementia (previously the 4^th^ ranked) was identified as 3^rd^ ranked with a different seed.

## Conclusion

Having at least one hospitalization and especially at least one potentially avoidable hospitalization was more prevalent among NH Black compared to NH White Medicare beneficiaries. This suggests a greater potential for prevention in primary care among NH Black patients. The findings further showed that focus on especially COPD in NH White and NH Black patients in primary care may be useful to limit potentially avoidable hospitalizations. Heart failure was another top contributors of potentially avoidable hospitalization among NH White respondents that was found to be a risk factor in multivariable regression.

## Supplementary Information

**Additional file 1.**

## Data Availability

Health and Retirement Study (HRS) public survey data are readily available for use to registered researchers on the HRS site: https://hrs.isr.umich.edu/about. With regard to restricted Medicare files for HRS respondents, users can apply for licensed access to these restricted data on the HRS site. Because analytic data files for this manuscript include restricted Medicare data, they are subject to data use agreements with the Centers for Medicare and Medicaid Services (CMS) and as such, not available for distribution. Interested researchers are encouraged to apply for use of these data on the HRS site. Unfortunately, the authors of the paper cannot distribute the data used for the analyses.

## Abbreviations

COPD: Chronic obstructive pulmonary disease
NH: Non-Hispanic
CMS: Centers for Medicare and Medicaid Services
HRS: The Health and Retirement Study
CCW: Chronic Condition Data Warehouse
IQR: Interquartile range
aORs: Adjusted odds ratios
CIs: Confidence intervals
ADRD: Alzheimer’s disease and related disorders/senile dementia
