# Supplementary material for: The importance of chronic conditions for potentially avoidable hospitalizations among non-Hispanic Black and non-Hispanic White older adults in the US: a cross-sectional observational study
Source: BMC Health Serv Res. 2022 Apr 9;22:468. doi: 10.1186/s12913-022-07849-y (PMC8994911; doi:10.1186/s12913-022-07849-y)
Supplement: Supplementary file 1 — Additional file 1. [file 12913_2022_7849_MOESM1_ESM.docx]

**SUPPLEMENTARY**


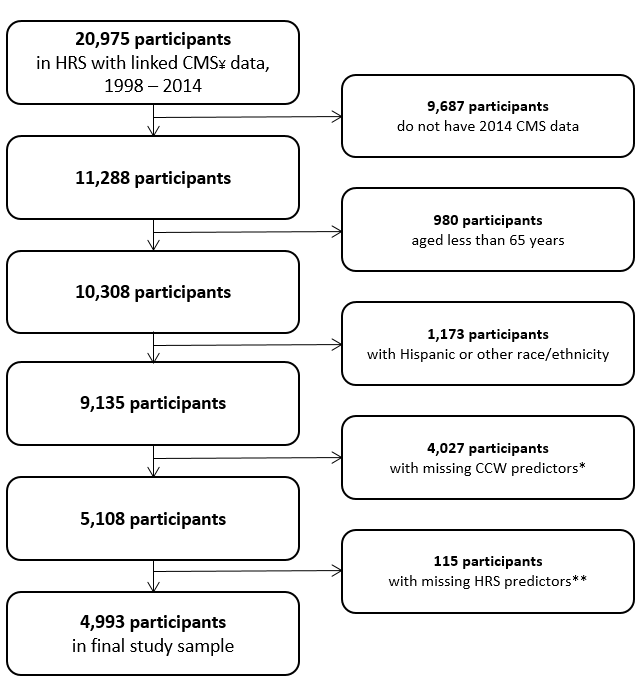


**Figure S1** Selection of study sample

*****Center for Medicare and Medicaid Services (CMS) predictors: Alzheimer’s disease, depression and somatic conditions were identified as missing if the beneficiary had not been enrolled for a sufficient amount of time in the Medicare fee-for-service program to identify the conditions based on the Center for Medicare and Medicaid Services’ Chronic Condition Data Warehouse (CCW) algorithms

******The Health and Retirement study predictors: sex, race/ethnicity, geographical region, education, wealth.

¥ CMS is the abbreviation of Centers for Medicare and Medicaid Services

| **Table S1** ICD-9 codes for potential avoidable hospitalizations | |
| --- | --- |
| **Disease categories** | **ICD-9 codes** |
| Chronic obstructive pulmonary disease, chronic bronchitis and asthma | 466–466.99, 490–490.99, 491.0, 491.1, 491.2, 491.20, 491.21, 491.8, 491.9, 492.0, 492.8, 493.00, 493.01, 493.02, 493.10, 493.11, 493.12, 493.20, 493.21, 493.22, 493.81, 493.82, 493.90, 493.91, 493.92, 494–494.99, 496–496.99 |
| Congestive heart failure | 98.91, 402.11, 402.91, 404.11, 404.13, 404.91, 404.93, 428.0, 428.1, 428.20, 428.21, 428.22, 428.23, 428.30, 428.31, 428.32, 428.33, 428.40, 428.41, 428.42, 428.43, 428.9, 518.4 |
| Constipation, fecal impaction, and obstipation | 564.0, 564.00, 564.01, 564.09, 560.39 |
| Dehydration, volume depletion including acute renal failure and hyponatremia, | 276.1, 276.5, 276.8, 584.0–584.99, 588.8, 588.81, 588.89, 588.9 |
| Hypertension and hypotension | 401.9, 402.10, 402.90, 403.10, 403.90, 404.10, 404.90, 458.0, 458.1, 458.2, 458.21, 458.29, 458.8, 458.9 |
| Poor glycemic control | 251.2, 250.2–250.29, 250.3–250.39, 250.1–250.19, 251.0, 250.02, 250.03, 790.29 |
| Seizures | 345–345.99, 436–436.99, 780.3– 780.39 |
| Urinary tract infection | 590.10, 590.11, 590.81, 590.9, 595.0, 595.1, 595.2, 595.4, 590.80, 595.89, 595.9, 597.0, 598.0–598.09, 599.0, 601–601.99 |
| Weight loss (failure to thrive) and nutritional deficiencies | 260–260.99, 261–261.99, 262– 262.99, 263–263.99, 268.0, 268.1, 783.21, 783.22, 783.3, 783.7 |

**Table S2 Sociodemographic characteristics of excluded respondents without 2014 CMS data***

| Characteristics | Hispanic  (n=651) | Non-Hispanic  White  (n=7,403) | Non-Hispanic  Black  (n=1,470) | Other/Missing  (n=163) | Overall  (n=9,687) |
| --- | --- | --- | --- | --- | --- |
| Age (years), mean (SD) | 77.8 (9.7) | 79.4 (9.0) | 76.7 (10.2) | 76.4 (9.2) | 78.9 (9.3) |
| Female, n (%) | 335 (51.5) | 3936 (53.2) | 838 (57.0) | 74 (45.4) | 5183 (53.5) |
| Wealth (USD), median (IQR) | 2.5 (0.0, 8.7) | 13.4 (3.2, 35.5) | 2.5 (0.0, 7.8) | 2.8 (0.0, 13.0) | 9.2 (1.5, 28.4) |
| Education (years), median (IQR) | 7.0 (3.0, 12.0) | 12.0 (10.0, 14.0) | 10.0 (7.0, 12.0) | 12.0 (7.0, 14.0) | 12.0 (9.0, 13.0) |

*Based on last HRS interview with a positive survey weight.
